# Subacute sclerosing panencephalitis, a measles complication, in an internationally adopted child.

**DOI:** 10.3201/eid0604.000409

**Published:** 2000

**Authors:** D J Bonthius, N Stanek, C Grose

**Affiliations:** Department of Pediatrics, University of Iowa, Iowa City, Iowa 52242, USA. daniel-bonthius@uiowa.edu

## Abstract

A healthy 13-year-old boy who had spent the first 4.5 years of his life in an orphanage in Thailand before adoption by an American couple became ill with subacute sclerosing panencephalitis and died several months later. The boy had most likely contracted wild-type measles in Thailand. Measles complications are a risk in international adoptions.


### Subacute Sclerosing Panencephalitis, a Measles Complication, in an Internationally Adopted Child

**Daniel J. Bonthius,* Nicholas Stanek,† and Charles Grose***
*Department of Pediatrics, University of Iowa, Iowa City, Iowa, USA;
†Department of Neurology, Medical Associates Clinic, Dubuque, Iowa, USA

A healthy 13-year-old boy who had spent the first 4.5 years of his life in an orphanage in Thailand before adoption by an American couple became ill with subacute sclerosing panencephalitis and died several months later. The boy had most likely contracted wild-type measles in Thailand. Measles complications are a risk in international adoptions.

Undiagnosed infections in internationally adopted children have been receiving increasing attention throughout the past decade. HIV, hepatitis viruses, *Treponema pallidum*, *Mycobacterium tuberculosis*, and intestinal parasites frequently complicate such adoptions (1,2). Subacute sclerosing panencephalitis (SSPE), a postinfectious neurologic complication of measles, can also occur. We describe a fatal case of SSPE in an internationally adopted child 9 years after he arrived in the United States.

### Case Report

A 13-year-old boy of Thai descent was referred to the pediatric neurology clinic at the University of Iowa Hospital with cognitive difficulties and a progressive movement disorder. The boy was born in 1984 and spent the first 4.5 years of his life in an orphanage in Thailand before being adopted by an American couple from Dubuque, Iowa. His adoptive parents were told by the adoption agency that the boy's medical history was unremarkable. No history of measles was reported. At adoption, the child appeared healthy and well nourished, and at no time afterwards did he have an illness suggestive of measles. Shortly after arrival, he displayed a short attention span and easy distractibility, for which he was eventually diagnosed with attention deficit hyperactivity disorder. He was treated with low-dose methylphenidate for several years with good results.

The child remained healthy throughout childhood until the age of 13 years 2 months, when his mother noted personality changes of irritability and worsened attention. Several months later, he developed intermittent, random, low-amplitude, lightning-like jerking movements of the extremities. The abnormal movements (thought to be tics) improved moderately, but transiently, after the methylphenidate was discontinued.

During the next several months, the boy became increasingly withdrawn and emotionally labile. He was treated for depression, but fluoxetine induced a marked worsening of the movement disorder and was discontinued. He was next treated with valproic acid, again with worsening in the movement disorder and no improvement in the psychiatric symptoms. Although the boy's academic performance had previously been average, he began to fail academically. He lost previous mathematics and language skills, and his teachers and parents noted progressive memory deficits. The movement disorder evolved from random myoclonic jerks of all four extremities to drop attacks many times a day, during which, while walking or standing, he would suddenly fall to the floor.

On examination at 13 years 9 months, the boy appeared healthy. He was alert and cooperative, but produced little spontaneous or prompted speech. He followed simple verbal commands, but had difficulty with more complex ones and appeared confused by simple written commands;

Address for correspondence: Daniel J. Bonthius, Division of Child Neurology, Department of Pediatrics, 2504 JCP, University of Iowa Hospital, 200 Hawkins Drive, Iowa City, IA 52242, USA; fax: 319-356-4855; e-mail: daniel-bonthius@uiowa.edu.

his adoptive mother indicated that at earlier times, he could have easily understood and followed such commands. Cranial nerve examination was notable for saccadic pursuit movements of gaze, hypometric saccades, and mild facial diplegia. Motor examination was notable for cogwheeling in the upper extremities bilaterally, especially on pronation-supination. The gait was remarkable for diminished bilateral arm swing. The posture and stance were remarkable for intermittent shocklike dipping of the head and shoulders with no apparent change in level of consciousness or postictal state.

Results of magnetic resonance imaging (MRI) (Figure 1) were focally abnormal with a single patch of increased T2 signal intensity and decreased T1 signal intensity in the subcortical white matter of the left frontal lobe. This focal lesion did not enhance with gadolinium. Results of an electroencephalogram (EEG) revealed high-amplitude bursts of periodic slow-wave complexes every 4–10 seconds, often accompanied by observable axial myoclonic spasms. The periodic slow-wave complexes arose from background activity that was essentially normal, except for some mild bifrontal dominant slowing (Figure 2). Cerebrospinal fluid (CSF) cytology, glucose, and total protein levels (15 mg/dL) were normal, but CSF immunoglobulin G (IgG) was elevated at 16.3 mg/dL (normal, 0.5–5.9 mg/dL). Measurement of specific antibodies by enzyme-linked immunosorbent assay revealed that rubeola (measles) IgG antibodies were markedly elevated in the CSF at 1:160 (normal, <1:5) and in the serum at 1:5120. Rubeola IgM antibody titers were undetectable in both CSF and serum. Both the EEG and CSF patterns were pathognomonic for SSPE and that diagnosis was made.

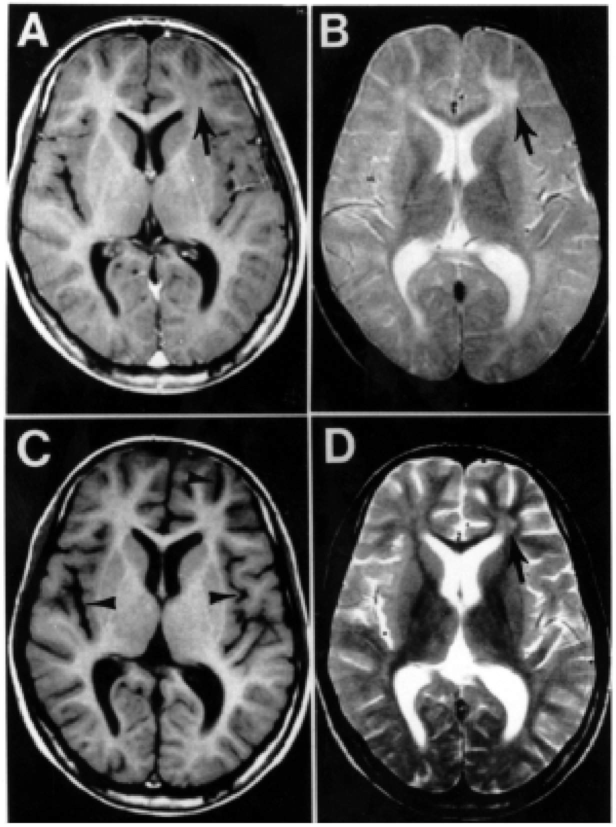

Figure 1. MRI scans of brain at time of presentation in the neurology clinic (A and B) and 3 months later (C and D). Panels A and C are T1-weighted images; B and D are T2-weighted images. The initial MRI scan (A and B) reveals a focal abnormality in the subcortical white matter of the left frontal lobe, consisting of a hypointense signal on the T1-weighted image (arrow in A) and a hyperintense signal on the T2-weighted image (arrow in B). In the follow-up scan, the focal abnormality in the left frontal lobe is less obvious than previously (arrow in D), but advanced and diffuse cortical atrophy is present, signified by the ventriculomegaly and markedly enlarged sulci (arrowheads in C).

The patient was placed on phenytoin, and the frequency of the drop attacks abated. Three months later, his neurologic status deteriorated rapidly, and he became obtunded. Repeat EEG again revealed high-amplitude, slow-wave complexes, but this time they arose from a diffusely and markedly slow background rhythm (Figure 2). Repeat MRI was most notable for advanced diffuse cortical atrophy that had not been present on the initial study. The focal abnormality in the subcortical white matter of the left frontal lobe was still detectable, but was less striking than initially (Figure 1). The patient died in June 1998, one week after the onset of acute deterioration. Permission for a postmortem study was denied.

### Conclusions

SSPE is a neurodegenerative disease caused by persistent infection of the brain by an altered form of the measles virus. Neither the biology underlying the viral persistence nor the triggering mechanism for viral reactivation is well understood. In most cases, infected children remain symptom-free for 6-15 years after acute measles infection (3).

Several factors suggest that this patient contracted measles in Thailand. First, the most consistent risk factor for SSPE is acquiring measles before the second birthday (4); this child

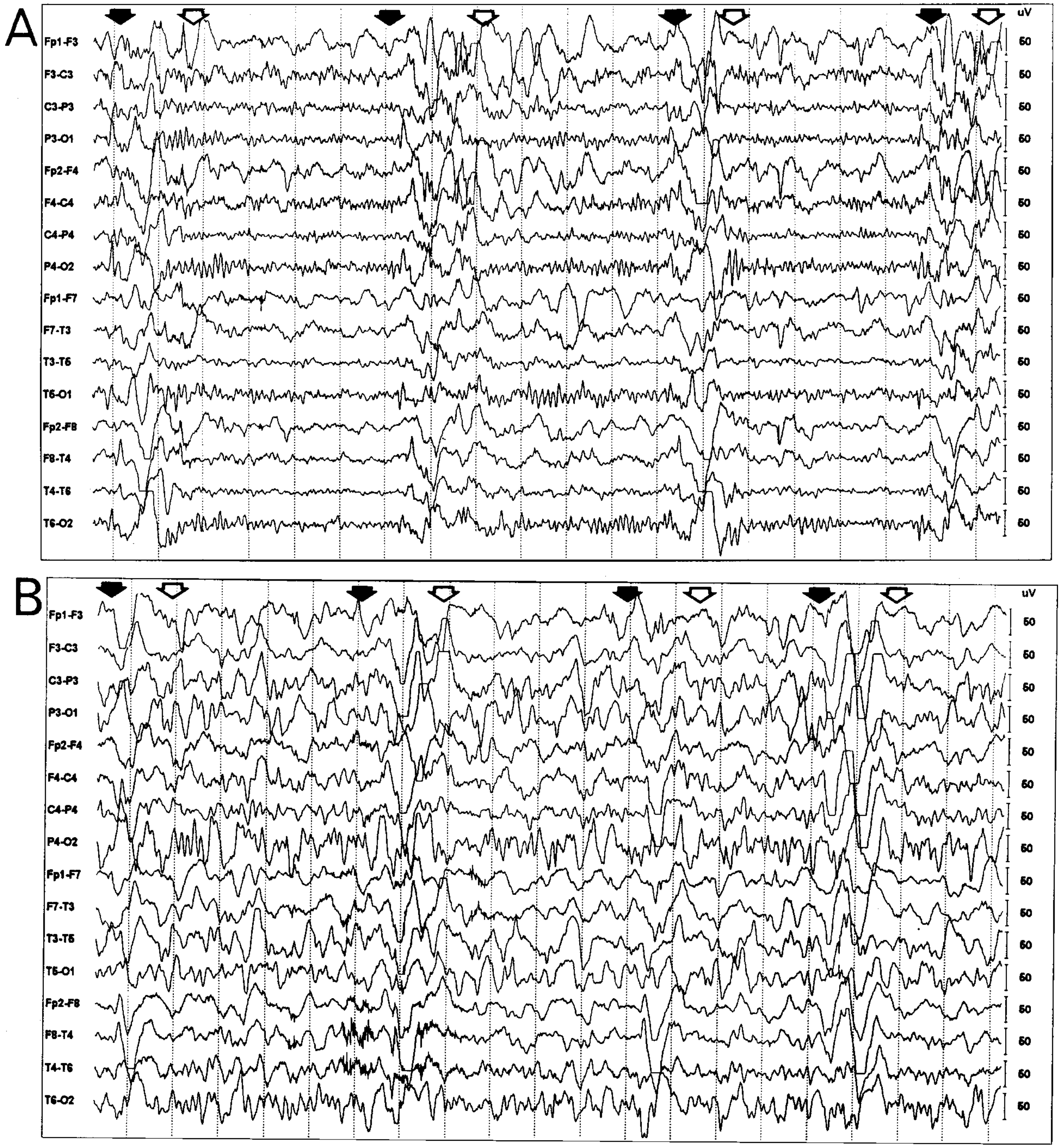

Figure 2. Electroencephalogram (EEG) at the time of presentation in the neurology clinic (A) and 3 months later (B). The initial EEG (A) reveals periodic bursts of high-amplitude, slow-wave complexes. (Onset of the complexes is indicated by solid arrows; offset, by open arrows.) The background rhythm is normal, except for bifrontal slowing. This "burst-suppression" pattern is highly characteristic of subacute sclerosing panencephalitis (4). EEG 3 months later, when the patient's clinical status has worsened (B), again shows periodic high-amplitude slow waves (again, between the solid and open arrows), but they now arise from a diffusely slowed background rhythm, which nearly obscures the periodic slow waves. In both A and B, the interval between each vertical dotted line is one second.

was still in Thailand at that age. Second, the incidence of SSPE in Asia is substantially higher than in North America; for example, the incidence is 2 per 100,000 population in India and 10 per 100,000 population in Pakistan, but only 1 per million population in the United States (4). Certainly, measles was endemic in Thailand when this patient lived there. Third, the number of cases of measles in Iowa during the nationwide outbreak in 1989–91 was relatively low; for example, there were no admissions for measles to the University of Iowa pediatrics ward during that period (unpublished data of authors). Finally, neither the parents nor the pediatrician noted any disease symptoms compatible with measles after the 4-year-old arrived in Iowa.

The initial symptoms of SSPE usually involve regressive changes in intellect and personality. Within several months, the psychological symptoms are compounded by neurologic ones, most often consisting of myoclonic jerks. A relentless mental and motor deterioration then ensues, culminating in extreme neurologic dysfunction and death within several years of the onset of symptoms (5). Our patient's clinical course reflected this typical natural history.

SSPE is accompanied by a unique set of laboratory abnormalities that facilitate its diagnosis. The persistent measles encephalitis induces a robust humoral immune response (6). Therefore, CSF in SSPE will typically have normal cellular components, glucose and total protein, but markedly elevated values of gammaglobulin (hyperglobulinorrachia greater than 20% of the total protein), and anti-measles antibodies (5,7). Typically, serum anti-measles antibody titers are also grossly elevated. In nearly all cases, EEG reveals a "burst-suppression" pattern at some point in the course of SSPE. This is one of two conditions in which a burst-suppression pattern is observed in a noncomatose patient. (The other condition is Creutzfeldt-Jakob disease.) The bursts of abnormal sharp and slow waves typically arise out of a normal background EEG activity early in the course of SSPE, but this background activity deteriorates to diffuse slow waves as the disease progresses (8). All of these characteristic immunologic and electrophysiologic abnormalities were observed in our patient. Because MRI came into clinical use after SSPE became rare in industrialized countries as a result of widespread measles vaccinations, few reports have been published on MRI findings in SSPE (9-11). The MRI profile of SSPE includes focal abnormalities in the subcortical white matter early in the course of disease and diffuse cerebral atrophy at later stages of the disease, as observed in our patient.

Further cases of SSPE are likely to occur among internationally adopted children. An increasing proportion of such children have spent their early childhood years institutionalized in crowded orphanages of Eastern Europe, Russia, and Asia, where conditions are fertile for outbreaks of measles and immunizations are often nonprotective (1-2). The incidence of SSPE among nonimmunized children is 100-200 times higher than among those who have been immunized effectively. (A recently reported case of SSPE in the United States involved an unimmunized child of Cambodian descent who contracted measles at the age of 1 year during the last outbreak in California in 1989 [12]. This child, who became ill with SSPE at age 9 years, had never traveled outside the United States and was not adopted.)

Because of the current widespread administration of measles vaccine in the United States, the incidence of acute measles infection has been dramatically reduced (13) and SSPE has been virtually eliminated and forgotten. However, measles, with the associated risk for SSPE in late childhood (14, 15), remains a recurrent public health hazard in developing nations.

Dr. Bonthius is a pediatric neurologist and director, Neuroteratology Laboratory, University of Iowa, where he conducts research on the injurious effects of environmental and infectious agents of the developing brain.

### References

1. Hostetter MK. Infectious diseases in internationally adopted children: the past five years. Pediatr Infect Dis 1998; 17: 517-8.
2. Hostetter MK. Infectious diseases in internationally adopted children: Findings in children from China, Russia, and Eastern Europe. Adv Pediatr Infect Dis 1999; 14: 147-61.
3. Gascon GG. Subacute sclerosing panencephalitis. Semin Pediatr Neurol 1996; 3:260-9.
4. Britt WJ. Slow viruses. In: Feigin R, Cherry J, editors. Textbook of pediatric infectious diseases. 4th ed. Philadelphia: W.B. Saunders Company, 1998; p. 646-65.
5. Dyken PR. Subacute sclerosing panencephalitis: current status. Neurol Clin 1985; 3:179-96.
6. Norrby E, Kristensson K. Measles virus in the brain. Brain Res Bull 1997; 44:213-20.

7. PeBenito R, Naqvi SH, Arca MM, Schubert R. Fulminating subacute sclerosing panencephalitis: Case report and literature review. Clin Pediatr 1997; 36:149-54.
8. Crowell J. Clinical neurophysiology: Video EEG studies. Symposium to ICNA/CNS Subacute Sclerosing Panencephalitis meeting: Update. San Francisco, CA, Oct 1, 1994.
9. Winer JB, Pires M. Kernode A, Ginsberg L, Rosser M. Resolving MRI abnormalities with progression of SSPE. Neuroradiology 1991; 33:178-80.
10. Anlar B, Saatci I, Kose G, Yalaz K. MRI findings in subacute sclerosing panencephalitis. Neurology 1996; 47:1278-83.
11. Geller TJ, Vern BA, Sarwar M. Focal MRI findings in early SSPE. Pediatr Neurol 1987; 3:310-12
12. Park SY. Subacute sclerosing panencephalitis in an identical twin. Pediatrics 1999; 104:1390-4.
13. Notifiable diseases/deaths in selected cities. MMWR Morb Mortal Wkly Rep 2000; 48:1183-90.
14. Assaad F. Measles: Summary of worldwide impact. Rev Infect Dis 1983; 5:452-9.
15. Moodley M. Subacute sclerosing panencephalitis in the developing world. S Afr Med J 1992; 82:72-3.
